# Four-Dimensional Echocardiography Is an Accurate Tool for Coronary Sinus Evaluation in Patients with Persistent Left Superior Vena Cava Diagnosis

**DOI:** 10.15190/d.2020.15

**Published:** 2020-12-09

**Authors:** Adina Glodeanu, Diana Alexandra Cherata, Radu Teodoru Popa, Didi Liliana Popa, Linda Barbulescu, Sorin Ioan Zaharie, Andreea Loredana Golli, Mihnea Valeriu Glodeanu

**Affiliations:** ^1^University of Medicine and Pharmacy of Craiova, Romania; ^2^University of Medicine and Pharmacy of Craiova, Calafat Municipal Hospital, Romania; ^3^Filantropia Municipal Hospital, Craiova, Romania; ^4^University of Craiova, Romania

**Keywords:** Cardiovascular imaging, multislice-computer tomography, persistent left superior vena cava, PLSVC, dilated coronary sinus, four-dimensional echocardiography; real-time three-dimensional echocardiography.

## Abstract

Persistent left superior vena cava (PLSVC) is a rare vascular congenital anomaly yet the most common for the thoracic venous system. Usually asymptomatic, PLSVC is commonly diagnosed when echocardiography or other cardiovascular imaging is performed. Due to venous drainage abnormality, PLSVC is frequently associated with other anomalies of the intrinsic heart’s conduction system, leading to tachy- or brady- arrhythmias. We present the case of a patient with 20 years history of supraventricular rhythm disorders diagnosed with isolated PLSVC. Furthermore, we discuss the diagnostic approach providing insights into four-dimensional echocardiography (4DE) evaluation for PLSVC diagnosis, assuming that there is a direct correlation between coronary sinus dilatation caused by abnormal venous return and supraventricular rhythm disorders. We highlight that correct understanding of the pathophysiology of PLSVC will lead to a reduction in unnecessary and potentially harmful testing, to a shorter diagnostic time and to a financial resource saving, as a whole.

## INTRODUCTION

Persistent left superior vena cava (PLSVC) is a rare vascular congenital anomaly, still the most common for the thoracic venous system^1,2^. It results from early cardiac development, when the left superior cardinal vein caudal to the brachiocephalic vein fails to regress. Despite PLSVC is a commonly isolated condition, associations between PLSVC and other cardiovascular abnormalities such as atrial septal defect, bicuspid aortic valve, aortic coarctation, coronary sinus ostial atresia and cor triatriatum (triatrial heart) were described in the literature^1^. Moreover, because of embryologic conductive tissue derangements, patients with congenital anomalous venous return are at emerging risk of manifesting various cardiac arrhythmias^1^. The presence of PLSVC may compromise catheter placement within the right side of the heart when left subclavicular vascular access is approached^3,4^. Usually asymptomatic, PLSVC is commonly diagnosed when echocardiography or other cardiovascular imaging is performed^5^. We present the case of a patient with 20 years history of supraventricular rhythm disorders diagnosed with isolated PLSVC and we discuss the diagnostic approach providing insights into the importance of noninvasive imaging techniques for PLSVC detection and evaluation.

## CASE REPORT

A 62-year-old hypertensive female patient known for repeated supraventricular rhythm disorders from 1998 (atrial fibrillation, atrial flutter, atrial tachycardia) and iatrogenic amiodarone induced hyperthyroidism, accusing palpitations and multiple episodes of irregular heartbeat with sudden onset at rest was sent to our clinic for cardiac evaluation.

At clinical examination the patient was normostenic, with a blood pressure of 120/60 mmHg and regular heart rate of 65 beats per minute, pale and marbled skin, xerostomia and xerophthalmia. Blood tests revealed normal values except of a mild hypochromic anemia. The patient was receiving efficient anticoagulation with an International Normalized Ratio (INR) of 2. INR calculation uses the division between patients’ determined prothrombin time and normal plasma prothrombin time using the International Sensitivity Index (ISI) as an exponent. The optimal INR range for efficient anticoagulation is between 2 and 3 for rhythm disorders patients.

Electrocardiogram (ECG) on admission showed regular sinus rhythm with heart rate of 65 bpm, PR interval of 140 msec, QRS axis at -45 degrees, QRS complex of 130 msec, QT interval of 400 msec, with no specific repolarization abnormalities. During hospitalization the patient developed multiple episodes of atrial tachycardia with a heart rate of 100 bpm. The ECG remained stable at discharge. Chest x-ray was normal.

**Figure 1:**
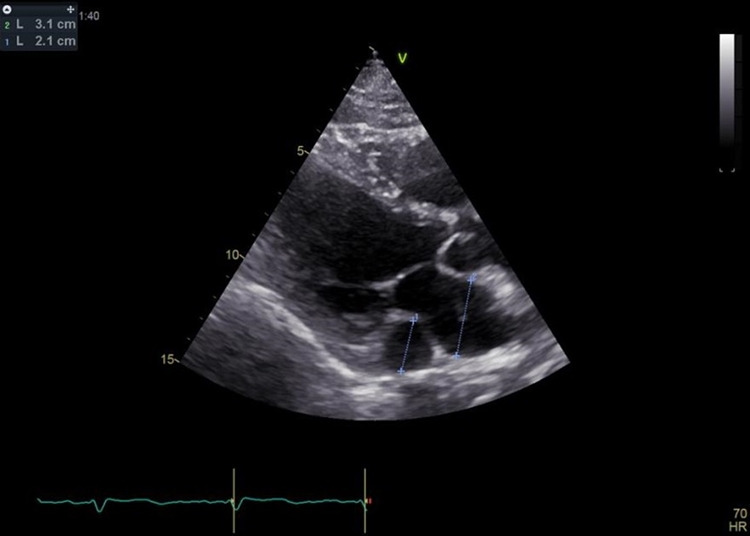
Two-dimensional transthoracic echocardiography, parasternal long axis view, illustrating dilated coronary sinus (L1-21mm diameter)

Two-dimensional (2D) transthoracic echocardio-graphy at presentation revealed trivial mitral regurgitation, slightly dilated left atrium, functional mild tricuspid regurgitation, mild reduced left ventricular ejection fraction (LVEF) and dilated coronary sinus with normal right sided filling pressures, raising the suspicion for the presence of PLSVC (Figure 1).

**Figure 2:**
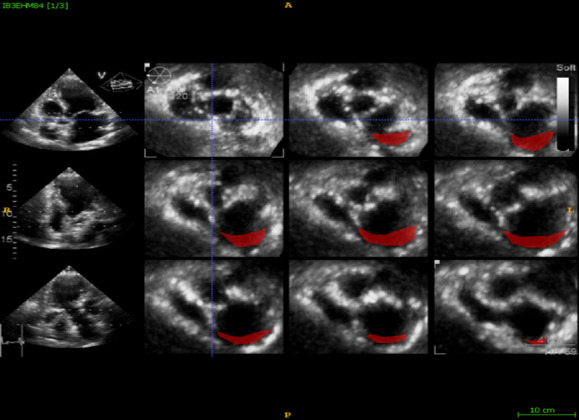
Four-dimensional echocardiography: frame by frame volume measurements of the dilated sinus

To better quantify coronary sinus dilatation, we performed four-dimensional echocardiography (4DE) measurements of the dilated coronary sinus in its last 5 cm emerging to right atrium, manually measuring frame by frame its volume (Figure 2).

Typically, the "bubble" test revealed the opacification of the coronary sinus when injecting the sparked serum into the left peripheral arm veins, suggesting the presence of PLSVC.

**Figure 3:**
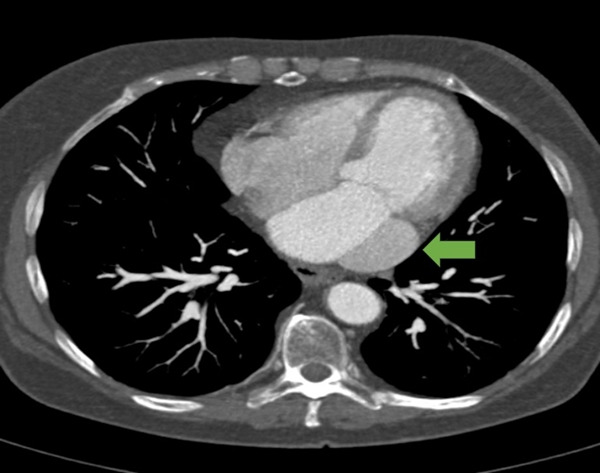
Multislice computer tomography, venous phase left atrium and dilated coronary sinus (green arrow)

**Figure 4:**
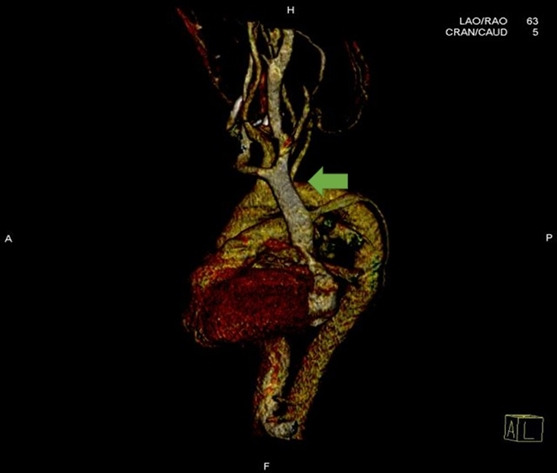
Computed tomography angiography, venous phase – three-dimensional view of persistent left superior vena cava (green arrow)

Multislice cardiac tomography (MSCT) was necessary for PLSVC evaluation showing normal thoracic aorta, brachiocephalic trunk, subclavicular arteries and common left carotid artery. In the venous phase we noted the presence of left superior cave vein and the brachiocephalic trunk and azygos vein openning to the left of the median line in the upper left cava, draining into the coronary sinus (Figure 3, Figure 4).

The electrophysiological study revealed sustained typical atrial flutter and nodal duality. Since the patient was under high doses of amiodarone and there was a functional block at the level of the cavotricuspidian isthmus, the radiofrequency ablation was postponed for a further date.

## DISCUSSION

In this paper we present a case of a patient with a long history of supraventricular rhythm disordersdiagnosed with isolated PLSVC. 

The presence of PLSVC may occur during the 8^th^ week of gestation when right and left superior cardinal veins anastomosis result in the brachiocephalic vein. If normal regression of the left superior cardinal vein to become "ligament of Marshall" fails to occur it results into a persistent left-sided vascular structure that drains into the coronary sinus^6^.

Typically, in the case of PLSVC being asymptomatic a random transthoracic echocardio-graphy reveals a dilated coronary sinus. However, dilation of the coronary sinus can commonly be observed in the case of conditions evolving with elevated right atrial pressure or coronary arterio-venous fistula, partial anomalous pulmonary venous return or a shunt flow between the left atrium and coronary sinus.

Coronary sinus dilation without any evidence of right sided high filling pressures, opacification of the dilated coronary sinus before the right atrium (RA) when contrast agent is injected into a left arm vein and opacification of the right atrium before the coronary sinus when injected into the right arm vein, are traditional echocardiography criteria used in clinical practice for PLSVC detection and diagnosis.

Usually MSCT or magnetic resonance investigations are used to rule out variations in the typical anomalous venous course along with transesophageal echocardiography^7^ or radionuclide angiocardiography.

For coronary sinus visualization and quantification both echocardiography and MSCT can be used, 4DE being able to quantify its volume apparently with similar results as MSCT^8^, but with significantly difference between the time of the procedure and radiation exposure. 4DE offers much faster and easier data acquisition, immediate display of anatomy and the possibility of online quantitative analysis of cardiac structures, such as the coronary sinus, using a single-acquisition data set, without a priori assumptions regarding its shape^9^ (Figure 2). 4DE is less operator-dependent than 2D echocardiography, allowing for more reproducible and objective echocardiographic assessment of the morphology and function of cardiac chambers and valves^9^.

In our case, we used 4DE and MSCT for coronary sinus assessment. According to the study of Conca et al.^8^ MSCT and 4DE agreed for coronary sinus systolic and diastolic measurements (Lin R = -0.09 [95% confidence interval 0.73-0.93]). We noticed that coronary sinus volumes measured by MSCT and 4DE are comparable, with a low difference between the two measurements (Table 1). We concluded that in PLSVC patients, it is possible to measure the volume of the dilated coronary sinus at its proximal segment using 4DE and that in this particular case its volume is ~24 times higher than normal volume values (27502 mm^3^ versus 1129 mm^3^).

**Table 1:** Volume comparison between a normal patient and a patient with a dilated coronary sinus

Diagnosis	PLSVC	Normal
*Age*	62 years	60 years
*Coronary sinus*	Dilates	Normal
*Volume of coronary sinus endsystole on computed tomography angiogram*	25379 mm^3^	1006 mm^3^
*Volume of coronary sinus endsystole on 4D ecocardiography *	27502 mm^3^	1129 mm^3^

Even though it has an asymptomatic evolution, PLSVC presence complicates the access to the right side of the heart or pulmonary vasculature via left subclavian vein during implantable device procedures such as permanent pacemaker and cardioverter defibrillator^10^. Regarding its arrhythmogenic implications, the epicardial space can be accesses via the coronary sinus for ablation of accessory pathways responsible for ventricular arrhythmia. In addition, coronary sinus musculature may form arrhythmogenic areas and atrioventricular accessory connections being part of macro or microreentrant atrial arrhythmias^11^. Moreover, abnormal electrophysiologic function can consequently emerge because of PLSVC, manifesting as both tachyarrhythmias (supraventri-cular tachycardias, atrial fibrillation/flutter or Wolff-Parkinson-White syndrome) and bradyarrhythmias (due to atrioventricular conduction blocks)^11,12^. Not least, arrhythmias can secondarily arise due to the abnormal anatomy and physiologic stresses placed on the conductive tissue that leads to right atrium enlargement or coronary sinus dilation^12^.

Therefore, a documented PLSVC forces an anamnesis about possible cardiac symptoms, such as decreased exercise tolerance, progressive fatigue, chest discomfort, palpitations or syncope and annual clinical follow-up, including an electrocardiogram and cardiac evaluation, should be continued^12^.

We consider that in our case there is a correlation between PLSVC diagnosis and supraventricular rhythm disorders caused by abnormal venous return. The correlation between isolated PLSVC, coronary sinus size and supraventricular rhythm disorders incidence in these patients is unknown and still current imaging methods cannot predict if patients with PLSVC would develop future supraventricular rhythm disorders.

## CONCLUSION

The presence of a dilated coronary sinus on echocardiography should alert the clinician towards the possibility of PLSVC. Its clinical implications regard not only left subclavian vein access to the right-side during device implant procedures but also abnormal electrophysiology function. The diagnosis of PLSVC includes multiple investigations: echocardiography, saline contrast echocardiography (bubble test) and MSCT. We have demonstrated that using real-time evaluation and three-dimensional echocardiography we can obtain a correct understanding of the pathophysiology of persistent left superior vena cava, assuming that there is a direct correlation between coronary sinus dilatation caused by abnormal venous return and supraventricular rhythm disorders. We highlight the fact that using 4D echocardiography in persistent left superior vena cava evaluation may lead to a reduction in unnecessary and potentially harmful testing, to a shorter diagnostic time and to a financial resource saving, as a whole. Future studies are needed to test whether coronary sinus dilation assessed by 4DE can be used as predictive factor for arrhythmias in patients with PLSVC.

## KEY POINTS

**◊ ***We assume that there is a correlation between PLSVC and supraventricular rhythm disorders, probably caused by abnormal venous return*.

**◊** *None of current imaging methods is able to predict if patients with PLSVC will develop supraventricular rhythm disorders in the future.*

**◊** *Coronary sinus dilatation can be quantified by measuring its volume of by either four-dimensional echocardiography or by multislice computer tomography.*

**◊** *Further research is needed to test if coronary sinus dilation evaluated by four-dimensional echocardiography can predict arrhythmias in patients with PLSVC.*

**◊** *Proper understanding of pathophysiology of PLSVC will reduce unnecessary and potentially harmful testing.*
